# Assessing potential drug-drug interactions between clofazimine and other frequently used agents to treat drug-resistant tuberculosis

**DOI:** 10.1128/aac.01583-23

**Published:** 2024-04-10

**Authors:** Allan Kengo, Firdaus Nabeemeeah, Paolo Denti, Ryan Sabet, Gifty Okyere-Manu, Pattamukkil Abraham, Lubbe Weisner, Modiehi Helen Mosala, Sibongile Tshabalala, Janine Scholefield, Juan Eduardo Resendiz-Galvan, Neil A. Martinson, Ebrahim Variava

**Affiliations:** 1 Division of Clinical Pharmacology, Department of Medicine, University of Cape Town, Cape Town, South Africa; 2 Perinatal HIV Research Unit (PHRU), University of the Witwatersrand, Johannesburg, South Africa; 3 Bioengineering and Integrated Genomics Group, Council for Scientific and Industrial Research, Pretoria, South Africa; 4 Johns Hopkins University Center for Tuberculosis Research, Division of Infectious Diseases, School of Medicine, Baltimore, Maryland, USA; 5 Department of Internal Medicine, University of the Witwatersrand, Klerksdorp/Tshepong Hospital Complex North-West Province, Klerksdorp-Tshepong, South Africa; Providence Portland Medical Center, Portland, Oregon, USA

**Keywords:** clofazimine, drug-drug interaction, pharmacokinetics, linezolid, levofloxacin, drug-resistant TB, modeling, NONMEM

## Abstract

Clofazimine is included in drug regimens to treat rifampicin/drug-resistant tuberculosis (DR-TB), but there is little information about its interaction with other drugs in DR-TB regimens. We evaluated the pharmacokinetic interaction between clofazimine and isoniazid, linezolid, levofloxacin, and cycloserine, dosed as terizidone. Newly diagnosed adults with DR-TB at Klerksdorp/Tshepong Hospital, South Africa, were started on the then-standard treatment with clofazimine temporarily excluded for the initial 2 weeks. Pharmacokinetic sampling was done immediately before and 3 weeks after starting clofazimine, and drug concentrations were determined using validated liquid chromatography-tandem mass spectrometry assays. The data were interpreted with population pharmacokinetics in NONMEM v7.5.1 to explore the impact of clofazimine co-administration and other relevant covariates on the pharmacokinetics of isoniazid, linezolid, levofloxacin, and cycloserine. Clofazimine, isoniazid, linezolid, levofloxacin, and cycloserine data were available for 16, 27, 21, 21, and 6 participants, respectively. The median age and weight for the full cohort were 39 years and 52 kg, respectively. Clofazimine exposures were in the expected range, and its addition to the regimen did not significantly affect the pharmacokinetics of the other drugs except levofloxacin, for which it caused a 15% reduction in clearance. *A posteriori* power size calculations predicted that our sample sizes had 97%, 90%, and 87% power at *P* < 0.05 to detect a 30% change in clearance of isoniazid, linezolid, and cycloserine, respectively. Although clofazimine increased the area under the curve of levofloxacin by 19%, this is unlikely to be of great clinical significance, and the lack of interaction with other drugs tested is reassuring.

## INTRODUCTION

The surge in the incidence of rifampicin/drug-resistant tuberculosis (DR-TB) is an obstacle to global efforts to eradicate TB (1). Unlike its susceptible form, DR-TB is more challenging to manage and requires a longer treatment duration (2). However, new regimens utilizing novel and repurposed antimycobacterial agents have greatly reduced treatment duration (3) and mortality attributed to DR-TB (4).

Over the past decade, there has been renewed interest in clofazimine, a fat-soluble riminophenazine dye that was initially used to treat leprosy (5), partly because clofazimine-containing regimens showed the potential to safely shorten the duration of DR-TB treatment (6, 7). Although clofazimine is not part of the current WHO-recommended BPaL/M regimen for DR-TB (8), it is categorized as a group B drug, used when levofloxacin/moxifloxacin, bedaquiline, or linezolid cannot (8). In 2019, the South African Department of Health recommended a shorter 9–11-month oral DR-TB regimen (9) comprising bedaquiline, clofazimine, levofloxacin/moxifloxacin, linezolid, high-dose isoniazid, pyrazinamide, and ethambutol. Terizidone was used whenever there was established fluoroquinolone resistance.

Clofazimine is highly lipophilic and exhibits significant duration-dependent accumulation in tissues like fat, muscle, and skin (10), leading to relatively low serum concentrations (11). Little is known about its metabolism, and it is largely excreted unchanged (12). *In vitro* research suggests that clofazimine may inhibit some cytochrome enzymes (13) and common membrane efflux transport proteins like P-glycoprotein and breast cancer resistance protein (BCRP) (14).

Isoniazid is an important component of drug-sensitive TB treatment (15) and is used at higher doses for DR-TB (2, 9). Upon ingestion, isoniazid is rapidly absorbed, undergoes significant first-pass metabolism (16), and is eventually eliminated in urine (16, 17). It is metabolized by N-acetyltransferase-2 (NAT2) (17) which is encoded by a highly polymorphic *NAT2* gene (18). Polymorphism of the *NAT2* gene has been extensively studied, resulting in the phenotypic classification of populations into slow, intermediate, and rapid acetylators (17, 19–21), which are closely linked to isoniazid plasma concentrations.

Linezolid and levofloxacin are WHO-recommended group A drugs for inclusion in DR-TB treatment regimens (8). Linezolid is an oxazolidinone antibiotic that is well absorbed upon oral administration (22), metabolized by cytochrome P450 2J2, 4F2, and 1B1 (23), and eliminated unchanged or as metabolites in urine (22). Levofloxacin is a broad-spectrum fluoroquinolone antibiotic used against many bacterial diseases (24), including DR-TB (8). It is rapidly absorbed upon oral administration (25), and its absorption is affected by food (25) and chelating agents (26). Levofloxacin undergoes limited metabolism and is mainly excreted unchanged by the kidneys through glomerular filtration and transporter-mediated tubular secretion (25, 27). Terizidone is a group B drug for DR-TB (8) which is rapidly hydrolyzed into cycloserine in the gut (28, 29). Cycloserine is a D-alanine analog that interferes with bacterial cell wall synthesis by inhibiting L-alanine racemase and D-alanyl alanine synthetase enzymes (30). It is rapidly absorbed from the gut (31), partly metabolized but mostly eliminated unchanged in urine (32).

Safe use of clofazimine-containing regimens requires an understanding of the nature and extent of pharmacokinetic interactions with co-administered DR-TB drugs. The aim of this study was to investigate the pharmacokinetic interactions between clofazimine and isoniazid, linezolid, levofloxacin, and cycloserine in adults with DR-TB.

## RESULTS

### Study population

Data were available for 27 adult participants of whom 19 (70%) were men and 23 (85%) were living with HIV. Their median age and weight were 39 years and 52 kg, respectively (Table 1), and their median estimated glomerular filtration rate was 89.3 mL/min. Visit 2 samples from 16 participants were assayed for clofazimine. All samples from 27 participants were assayed for isoniazid. Linezolid and levofloxacin concentrations were assayed for 21 participants, and samples from six participants were assayed for cycloserine. For isoniazid, 5 (19%), 13 (48%), and 9 (33%) participants were categorized as slow, intermediate, and rapid acetylators, respectively.

**TABLE 1:** Participant baseline characteristics

Characteristics	Drug			
	Clofazimine	Isoniazid	Linezolid/levofloxacin	Cycloserine
Number of participants	16	27	21	6
Number (%) of male participants	10 (63)	19 (70)	13 (62)	4 (67)
Number (%) of participants living with HIV	14 (88)	23 (85)	16 (76)	5 (83)
Median (range) age (yr)	40 (20–66)	39 (20–62)	39 (27–68)	39 (33–53)
Median (range) weight (kg)	52 (35–66)	53 (37–66)	52 (37–74)	53 (55–70)
Median (range) fat-free mass (kg)	39.5 (25.7–51.9)	44.2 (26.0–56.5)	41.5 (26.0–51.6)	42.0 (35.7–51.6)
Median (range) height (m)	1.65 (1.30–1.76)	1.67 (1.30–1.81)	1.64 (1.49–1.80)	1.64 (1.56–1.79)
Median (range) creatinine clearance (mL/min)^*a*^		89.3 (35.0–143)	81.8 (50.5–136)	96.5 (71.4–115)
Median dose (range) (mg/kg)^*c*^	1.90 (1.52–2.86)	10.0 (7.35–12.1)	11.5 (8.11–16.2)/19.1 (13.5–26.3)	13.2 (10.7–17.6)^*b*^
Number (%) of participants with NAT2 acetylator status				
Slow		5 (19)		
Intermediate		13 (48)		
Rapid		9 (33)		

^*a*^ Creatinine clearance was calculated using the Cockcroft and Gault formula (33).

^*b*^ The dose of terizidone (cycloserine) was split into 250 mg and 500 mg in the morning and evening, respectively.

^*c*^ The weight normalized dose was calculated by dividing total daily dose of the drug by weight of the participant.

### Population pharmacokinetic analysis

**Fig 1:**
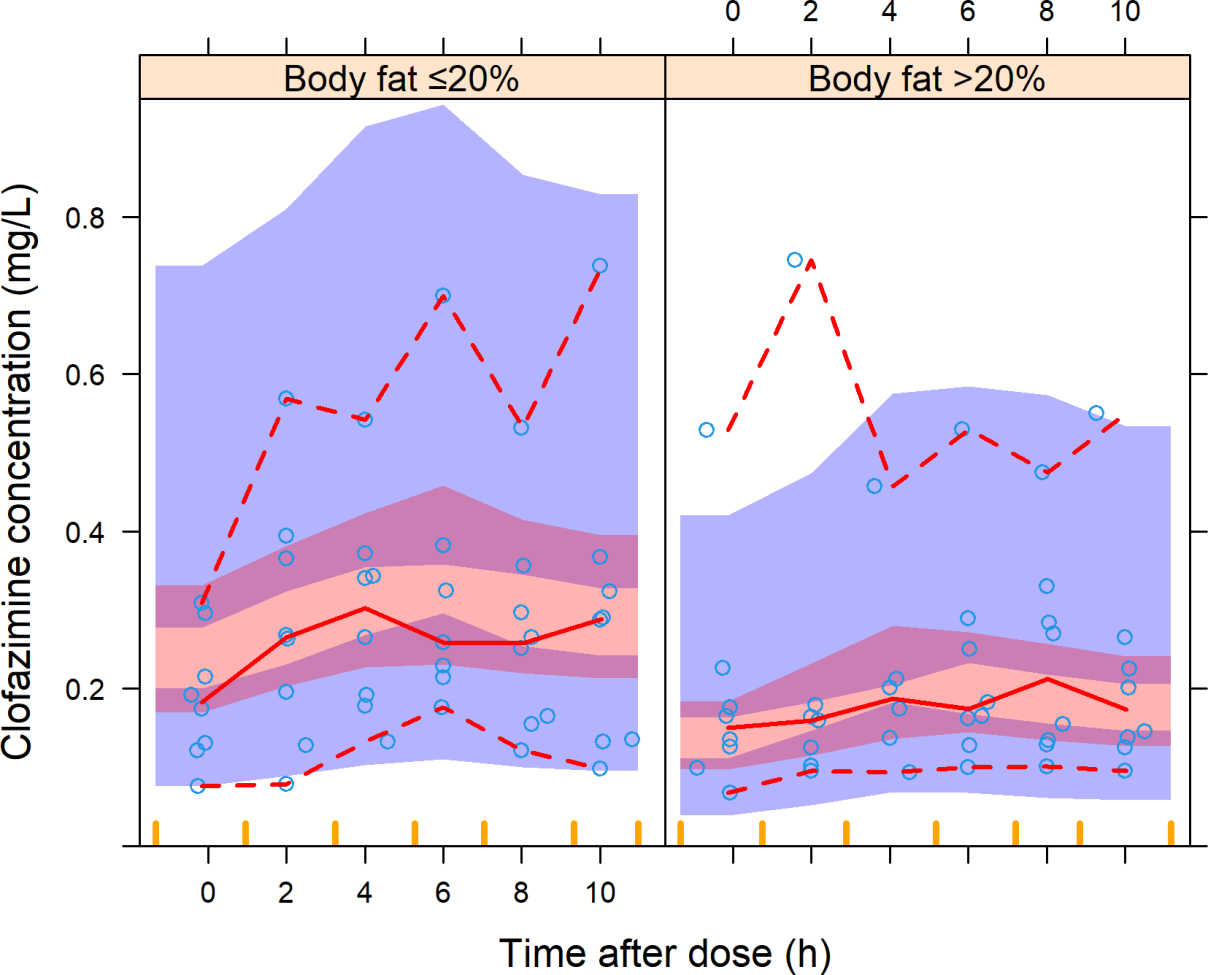
A visual predictive check (VPC) of clofazimine concentration versus time after dose, stratified by proportion of body weight that is fat. The solid and dashed lines represent the 5th, 50th, and 95th percentiles of the observed data (open circles), while the shaded areas represent the model-predicted 95% confidence intervals for the same percentiles. Of note, this VPC was obtained without re-estimating parameters (maxeval = 0 in NONMEM) which is like an external validation. CFZ, clofazimine.

Clofazimine data were adequately predicted by a previously published three-compartment disposition model (34), without re-estimating any pharmacokinetic parameter. In keeping with the previous model, exposure reduced with increasing proportion body fat (Fig. 1).

**Fig 2:**
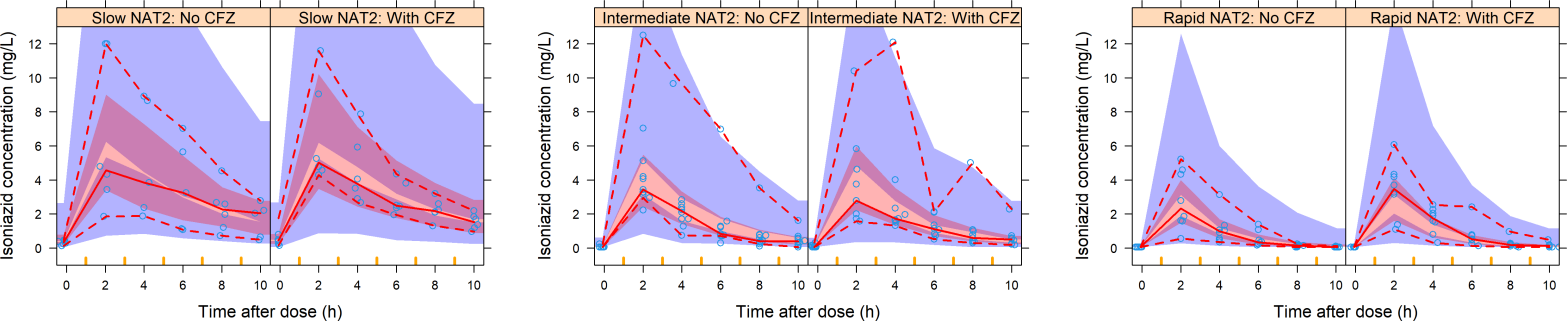
Visual predictive check of the isoniazid concentration versus time after dose, stratified by N-acetyltransferase-2 phenotype and study visit (with versus without clofazimine). The model without an effect of clofazimine fits all data on both study visits. The solid and dashed lines represent the 50th, 5th, and 95th percentiles of the observed data (open circles), while the shaded areas represent the model-predicted 95% confidence intervals for the same percentiles. NAT2, N-acetyltransferase activity and CFZ, clofazimine.

The typical parameter estimates for isoniazid, linezolid, and levofloxacin, and their 95% confidence intervals (95% CIs) are presented in Table 2. A two-compartment model with first-pass effect through a liver compartment (20) adequately described isoniazid data (Fig. 2). Fixing the typical absorption parameters to literature values (20), we estimated an intrinsic clearance of 12.2, 24.9, and 46.7 L/h for slow, intermediate, and rapid acetylators, respectively [change in objective function value (ΔOFV) = −39.0, 2 degrees of freedom (df), *P* < 0.001]. Inclusion of allometric scaling by fat-free mass (FFM) on all disposition parameters improved the fit (ΔOFV = −1.42). Between-visit variability (BVV) in clearance improved the model (ΔOFV = −14.2, 1 df, *P* < 0.001), but the 10% decrease in clearance we found on visit 2 was not significant (ΔOFV = −3.52, 1 df, *P* = 0.061).

**TABLE 2:** Table of model pharmacokinetic parameter estimates

Parameter	Typical parameter estimates (95% CI^*c*^)		
	Isoniazid	Linezolid	Levofloxacin
Clearance (L/h)^*a*^	–	3.04 (2.74–3.87)	6.81 (6.05–7.63)
Intrinsic clearance (slow acetylators) (L/h)^*a*^	12.2 (9.53–14.5)	–	–
Intrinsic clearance (intermediate acetylators) (L/h)^*a*^	24.9 (19.0–27.0)	–	–
Intrinsic clearance (rapid acetylators) (L/h)^*a*^	46.7 (37.9–56.4)	–	–
Volume of distribution (central compartment) (L)^*a*^	57.0 (49.0–61.9)	38.7 (37.0–43.1)	98.5 (90.3–107)
Inter-compartmental clearance (L/h)	1.70 (0.806–2.27)	–	–
Volume of distribution (peripheral compartment) (L)^*a*^	42.4 (9.88–52.9)	–	–
Bioavailability, F (fraction)^*b*^	1 (fixed)^*d*^	1 (fixed)	1 (fixed)
Absorption rate constant, ka (/L)	3.28 (fixed)^*e*^	1.14 (0.768–1.77)	2.05 (1.19–3.25)
Mean absorption transit time, MTT (h)	0.122 (fixed)^*e*^	0.928 (0.713–1.36)	1.32 (1.00–1.61)
Transit compartments, NN (n)	2.32 (fixed)^*e*^	12.6 (9.98–37.0)	15.7 (9.91–27.3)
Hepatic blood flow rate, QH (L/h)^*a*^^,^^*f*^	72.4 (fixed)	–	–
Unbound fraction, fu (%)^*f*^	95 (fixed)	–	–
Scaling factor on BOV for unobserved dose (fold change)^*h*^	–	1.60 (1.07–2.67)	1.99 (1.40–2.92)
Effect of CL_CR_ (+10 mL/min change) on CL (%)	–	–	+4.70 (+1.58 to +7.77)
Effect of clofazimine on clearance (%)	–	–	−14.8 (−23.2 to −5.88)
Additive error (mg/L)	0.021, i.e., 20% LLOQ^*i*^ (fixed)	0.284 (0.142–0.596)	0.238 (0.121–0.375)
Proportional error (%)	24.4 (21.1–28.0)	7.31 (5.36–8.59)	4.97 (3.81–6.01)
Variability (%CV)^*g*^			
BSV in clearance	16.2 (8.10–26.1)	31.6 (13.2–41.5)	18.3 (13.2–24.0)
BVV in clearance	18.4 (11.5–25.0)	33.3 (25.4–49.2)	17.0 (9.69–22.0)
BOV in ka	94.3 (4.27–101)	104 (74.5–127)	118 (88.9–156)
BOV in MTT	129 (87.0–215)	73.8 (47.4–101)	44.4 (31.6–56.0)
BOV in F	47.5 (34.3–54.6)	12.4 (6.48–18.4)	21.5 (16.5–27.9)

^*a*^ All clearance and volume parameters for isoniazid, linezolid, and levofloxacin were scaled with allometric scaling based on fat-free mass. The values reported here refer to a typical participant with a fat-free mass of 42 kg and total body weight of 52 kg.

^*b*^ Since only oral data were available, we set the typical bioavailability to the reference value of 1 and estimated variability around it.

^*c*^ Values in parentheses are empirical 95% conﬁdence intervals obtained by sampling importance resampling procedure.

^*d*^ For isoniazid, the typical bioavailability reported is pre-hepatic.

^*e*^ The typical values of the absorption parameters (ka, MTT, NN) of isoniazid have been fixed to those reported by Gausi et al. (20).

^*f*^ The isoniazid unbound fraction and hepatic flow rate were fixed to literature values (20, 35). The hepatic flow rate reported here is for a typical participant with a fat-free mass 42 kg.

^*g*^ Parameter variability was modeled as either between‑subject (BSV), between-occasion (BOV), or between‑visit (BVV) variability. Variability was assumed to be log-normally distributed and is reported here as the percent coefficient of variation (%CV) calculated by $\%CV=\sqrt{\omega^{2}}\;\times 100$.

^*h*^ This is a multiplicative factor increasing the BOV of absorption parameters for pre-dose concentrations following an unobserved dose.

^*i*^ LLOQ, lower limits of quantification. –, not applicable.

**Fig 3:**
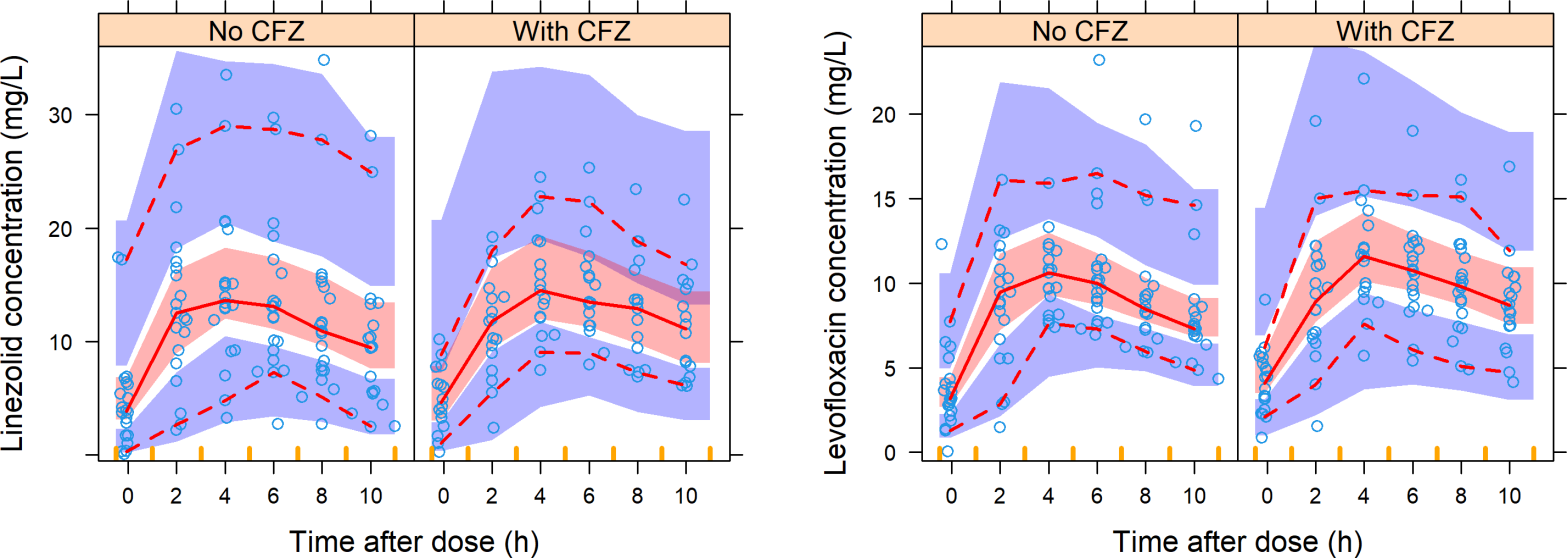
Left, VPC of linezolid concentration versus time after dose (stratified by study visit) showing that the model without effect of clofazimine fits the data on both visits. Right, VPC of levofloxacin concentration versus time after dose (stratified by study visit) showing that the model with an effect of clofazimine on clearance fits the data. The solid and dashed lines represent the 50th, 5th, and 95th percentiles of the observed data (open circles), while the shaded areas represent the model-predicted 95% confidence intervals for the same percentiles. CFZ, clofazimine.

Linezolid data were well described by a one-compartment model with first-order elimination and absorption through transit compartments (ΔOFV = −108, 2 df, *P* < 0.001) (Fig. 3). With allometric scaling by FFM included on disposition parameters (ΔOFV = −4.82), we estimated a typical clearance and volume of 3.04 L/h and 38.7 L, respectively. BVV in clearance improved the model (ΔOFV = −32.4, 1 df, *P* < 0.001), and we observed a non-significant (ΔOFV = −3.7, 1 df, *P* = 0.054) 2% decrease in clearance on visit 2. Additionally, a 1.6-fold increase in between-occasion variability (BOV) of the absorption parameters of unobserved pre-doses (ΔOFV = −6.2, 1 df, *P* = 0.013) significantly improved the model.

Levofloxacin data were well characterized by a one-compartment model with first-order elimination and transit compartment absorption (ΔOFV = −198, 2 df, *P* < 0.001) (Fig. 3), and the typical values of clearance and volume were 6.81 L/h and 98.5 L, respectively. Allometric scaling of disposition parameters by FFM improved the model (ΔOFV = −17.6). Renal function improved the fit (ΔOFV = −4.53, 1 df, *P* = 0.033), predicting a 4.70% (95% CI: 1.58–7.77) increase in levofloxacin clearance for every 10 mL/min increase in creatinine clearance from the median. At visit 2, when clofazimine was given, clearance was 15% (95% CI: 10–28) slower (ΔOFV = −10.7, 1 df, *P* = 0.001), and consequently the median area under the curve (AUC) increased from 140 to 166 mg·h/L (Fig. S3). An approximately twofold increase in variability of absorption parameters of the unobserved doses improved the model significantly (ΔOFV = −6.34, 1 df, *P* = 0.012), and so did BVV on clearance (ΔOFV = −4.3, 1 df, *P* = 0.038).

**Fig 4:**
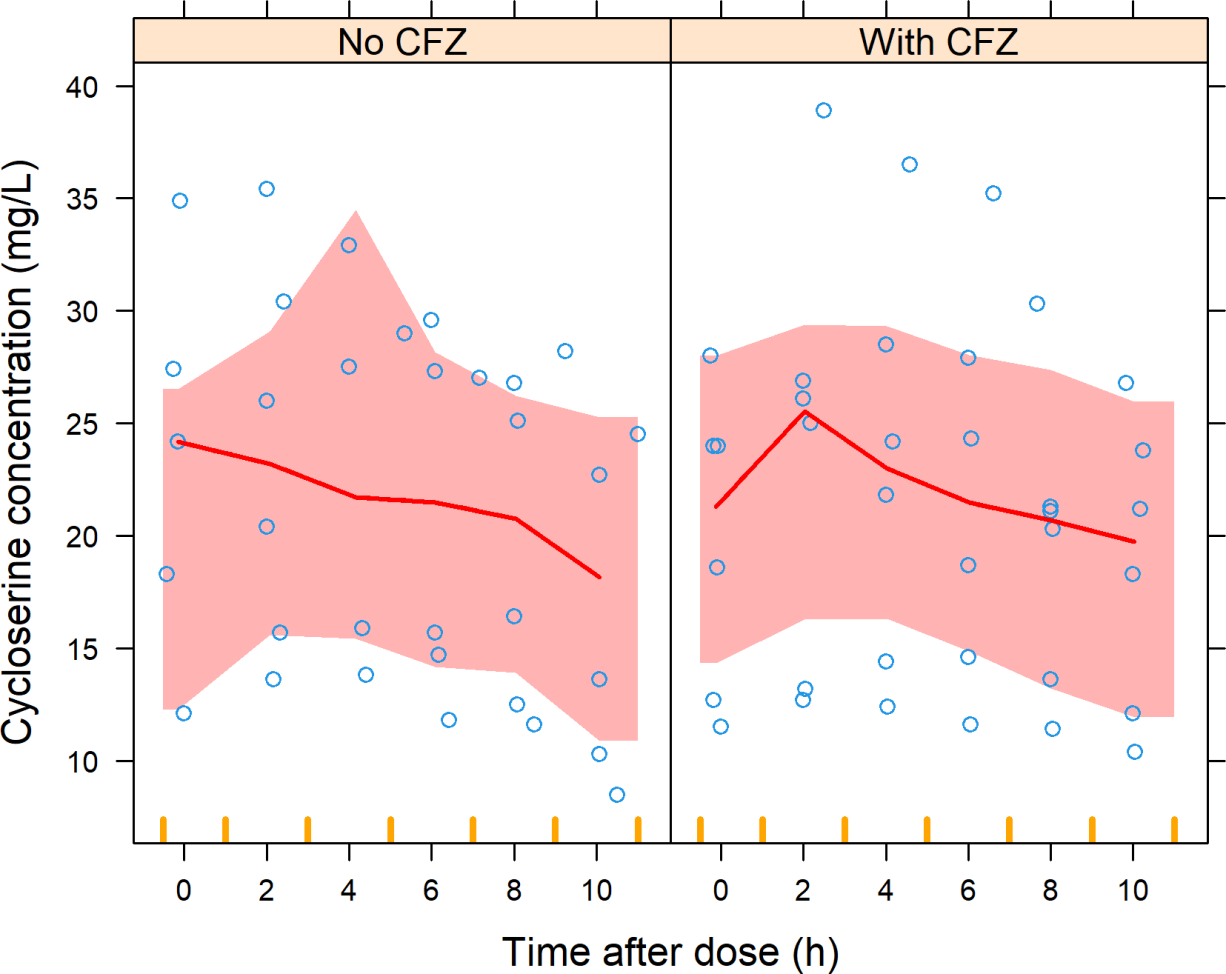
VPC of cycloserine concentration versus time after dose (stratified by study visit). The solid line represents the median of the observed data (open circles), while the shaded areas represent the model-predicted 95% confidence interval for th median. CFZ, clofazimine.

For cycloserine, we employed the one-compartment model with first-order absorption and elimination split into renal and non-renal clearance developed by Chirehwa et al. (29). While keeping all other parameters fixed, we re-estimated total clearance to account for differences between our data set and the original model. This was not significant (ΔOFV = −2.16, 1 df, *P* = 0.142), and neither was estimating different clearances during the two study visits (ΔOFV = −2.21, 2 df, *P* = 0.331) (Fig. 4).

For isoniazid, linezolid, and cycloserine, simulations showed that our samples of 27, 21, and 6 participants had 97%, 90%, and 87% power, respectively, to detect a 30% decrease in clearance across the two study visits.

## DISCUSSION

This is the first study we are aware of that has assessed the pharmacokinetic interaction between clofazimine and other DR-TB drugs. We found a 19% increase in levofloxacin AUC during visit 2, when clofazimine was added to the DR-TB regimen. However, we found no effect for high-dose isoniazid, linezolid, or terizidone/cycloserine.

Clofazimine exposures in our cohort were in line with predictions by a model developed by Abdelwahab et al. (34) in a similar population. Using this model, we confirmed the effect of body fat composition on clofazimine disposition by stratifying our data by proportion of body fat and showing that clofazimine plasma exposure reduces with increasing proportion of body fat. This is consistent with clofazimine’s known high lipophilicity and extensive fat distribution (6, 11, 34).

Our isoniazid model is structurally like the one by Gausi et al. (20). We estimate comparable increases in intrinsic clearance for intermediate and rapid acetylators compared to slow acetylators, consistently with the trimodal pattern of isoniazid acetylation due to genetic differences in NAT2 enzyme activity (16–20). We did not find significant changes in isoniazid pharmacokinetics after addition of clofazimine to the DR-TB regimen. This is not unexpected, since clofazimine has not been reported to affect NAT2 enzyme activity, the most important factor for isoniazid excretion (16, 17, 36).

Our linezolid pharmacokinetics results are consistent with previous findings (37, 38), and we found no statistically significant interaction with clofazimine. The absence of a pharmacokinetic interaction between linezolid and clofazimine is not unexpected as linezolid excretion has not been reported to be affected by clofazimine (22). Notably, we found slower absorption of linezolid than previously reported (37, 38), most likely due to drug intake with a meal (39).

Like Canouï et al. and Sidamo et al. (40, 41), a one-compartment model adequately described our levofloxacin data. This differs from the two-compartment model found by Denti et al. and Garcia-Prats et al. (42, 43), probably due to different sampling schedules and study populations. Our finding of a significant effect of creatinine clearance on levofloxacin clearance is in line with previous reports (24, 25, 40). We also found slower absorption of levofloxacin than has been previously reported (41–43), likely due to a food effect (24, 25).

Interestingly, we found a statistically significant 15% reduction in levofloxacin clearance during visit 2 when clofazimine was added to the DR-TB regimen. Although, no precise mechanism has been elucidated for the interaction between levofloxacin and clofazimine, *in vitro* studies have shown that clofazimine may inhibit P-glycoprotein and BCRP (14), which may be involved in the active tubular secretion of levofloxacin in the kidneys (26, 27). These transporters are located along the apical membrane of kidney tubules (44) and were found to efflux quinolone antibiotics into urine (27, 45). Their inhibition, as was found for ciprofloxacin (46), may be the reason behind this observed reduction in levofloxacin clearance when administered with clofazimine. The resulting increase in levofloxacin AUC, however, may not be of great clinical relevance as the exposures largely remained within the range of previous reports (25, 26, 47, 48).

For terizidone/cycloserine, the model by Chirehwa et al. (29) adequately described our data. The lack of a significant difference in total clearance across study visits, coupled with the fact that cycloserine is mostly cleared unchanged in urine (29, 32), suggests minimal likelihood of a major pharmacokinetic interaction between cycloserine and clofazimine.

One limitation of our study was the opportunistic nature of sampling during study visits, lacking strict observance of dosing prior to the pharmacokinetic visits and restrictions on food intake following the observed dose. Consequently, there was greater uncertainty in the pre-dose concentrations, which we addressed by allowing for greater variability in absorption parameters of unobserved doses. Similarly, food ingestion predictably led to slower absorption of linezolid and levofloxacin among our cohort. However, this had minimal impact on our primary findings, as the clearance parameters closely aligned with previous reports.

The study’s observational nature also resulted in a small sample size for terizidone as few participants in our cohort were on the long (terizidone-containing) regimen. We compensated for this by using a fixed literature model and only estimating separate clearance values for the two visits. Although simulations demonstrated acceptable power to rule out a large interaction, the results should be interpreted cautiously because the fixing of all pharmacokinetic parameters in the model may be an oversimplification.

Secondly, due to budget constraints, fewer participant samples were analyzed for clofazimine. This, however, had less consequence for the conclusions we drew about clofazimine as we only aimed to check if exposures in our cohort were in line with previous reports. Similarly, our sampling schedule did not include a 1-h post-dose sample and therefore was not sufficient to describe the rapid absorption of isoniazid. We mitigated this by fixing the typical absorption parameters to those reported by Gausi et al. (20). Finally, because of the long half-life of clofazimine, it was not possible to design a cross-over study. Consequently, we cannot exclude that any differences in clearance between the two study visits may be due to factors other than the delayed introduction of clofazimine interaction, like patient clinical improvement.

In conclusion, our data provide reassuring evidence that clofazimine does not cause significant pharmacokinetic interactions with other drugs commonly co-prescribed for DR-TB.

## MATERIALS AND METHODS

### Participants and study treatments

We conducted a non-interventional, prospective cohort study among participants with DR-TB about to start clofazimine-based treatment, at Klerksdorp/Tshepong Hospital in North-West Province, South Africa.

Newly diagnosed non-pregnant adults with confirmed pulmonary DR-TB, due to the start of clofazimine-containing treatment were recruited. As clofazimine has a delayed onset of action, the attending doctor assessed whether withholding it in the first 2 weeks of treatment would unduly harm the patient. If there was such an indication, patients were ineligible for inclusion. Other exclusion criteria were, isoniazid mono-resistance, poor prognosis at the time of enrolment, bedaquiline or clofazimine treatment in the previous 2 years, and refusal to test for HIV.

All participants were hospitalized for the first 2 weeks of treatment, receiving their TB treatment under direct observation, routinely accompanied by a meal. The first pharmacokinetic sampling (visit 1) was done on day 14. Clofazimine was then added to the regimen of all participants, and a second pharmacokinetic sampling (visit 2) was done 21 days later. At both visits, blood samples were drawn before, and then 2, 4, 6, 8, and 10 h after the observed dose. The daily oral doses for the drugs were as follows: 100 mg for clofazimine, 450/600 mg for high-dose isoniazid, 600 mg for linezolid, 750/1,000 mg for levofloxacin, and 750 mg for terizidone.

After collection, blood samples were immediately placed on ice, transferred to an on-site laboratory, and centrifuged at 1,500 × *g* to separate plasma. The plasma was aliquoted and stored at –80°C until batched transfer for analysis at the University of Cape Town’s Division of Clinical Pharmacology laboratory.

### Drug assays

Visit 2 samples in the first participants were assayed for clofazimine, and other drugs were assayed opportunistically depending on participants’ prescribed regimens as summarized in Fig. S1. Concentrations of pyrazinamide and ethambutol were not determined as they are unlikely to interact with clofazimine (36). Similarly, bedaquiline was not investigated because its long terminal half-life (49) and slow accumulation between the two study visits would make it difficult to interpret the data. Plasma concentrations were quantified using previously published validated liquid chromatography-tandem mass spectrometry assays; the lower limits of quantification (LLOQ) were 0.00781 mg/L for clofazimine (34), 0.105 mg/L for isoniazid (50), 0.100 mg/L for linezolid (37), 0.0781 mg/L for levofloxacin (42), and 0.313 mg/L for cycloserine (51), as previously described. More details are presented in Table S1 of supplementary materials.

### NAT2 phenotype

Consent for genetic studies was obtained, and blood samples were collected to determine the NAT2 genotype of participants. Genomic DNA was isolated as previously described (52), and genotyping was performed using Sanger sequencing of the *NAT2* gene after amplification using the following primers: 5′ ATTAACTGACATTCTTGAGC 3′ and 5′ GCACATAAGTTGATAATTAG 3′. Acetylator status was subsequently allocated based on the genotype of following four single nucleotide polymorphisms (SNPs): rs1801279 (c.191G>A), rs1801280 (c.341T>C), rs1799930 (c.590G>A), and rs1799931 (c.857G>A) (20, 53, 54). Participants were categorized as rapid acetylators if they were homozygous for the common allele of all four SNPs (i.e., GG, TT, GG, GG, respectively). Those who were heterozygous for only one of the four SNPs were categorized as intermediate acetylators, and those who were heterozygous for two or more SNPs, or homozygous for the variant allele for any of the SNPs were categorized as slow acetylators (20, 21).

### Pharmacokinetic modeling

Nonlinear mixed-effects modeling in the software NONMEM v7.5.1 (55) was used to analyze the data of all drugs. Perl-speaks-NONMEM v5.2.6, Pirana v3.0.0, and Xpose in RStudio were used to support model development (56).

First, clofazimine exposures in the first 16 participants were evaluated by fitting their visit 2 clofazimine concentrations to a previously published model (34) without estimation. Thereafter, suitable models were developed to characterize the pharmacokinetics of isoniazid, linezolid, levofloxacin, and cycloserine. One-compartment and two-compartment disposition models with first-order absorption and either lag or transit compartment absorption were evaluated (57). First-order elimination was explored for all drugs, and a well-stirred liver model with hepatic extraction was tested for isoniazid (20, 58).

To account for body size, allometric scaling with total body weight or fat-free mass was evaluated on all clearance and volume parameters with fixed exponents of 0.75 and 1, respectively (59). The effect of clofazimine co-administration was explored as a categorical covariate on clearance and bioavailability for all the drugs. Other previously reported covariates like NAT2 acetylator status for isoniazid, known drug interactions, and estimated creatinine clearance [Cockcroft and Gault formula (33)] were also explored during the covariate analysis.

Random effects on pharmacokinetic parameters were added at different levels: between-subject variability, BVV, and BOV (60), assuming a lognormal distribution. BOV was tested for all absorption parameters considering each dose as a separate occasion, while BVV was tested only on clearance between the two study visits. Residual unexplained variability was modeled by including both proportional and additive components with the lower bound of the additive component constrained to at least 20% of LLOQ (42). Data below the LLOQ were imputed to LLOQ/2 (48) as in Beal’s M6 method (61), and the additive component of their residual error was inflated by LLOQ/2 (20, 42).

Model development was guided by ΔOFV (62), which was assumed to follow *χ*^2^ distribution (for 1 df, ΔOFV > 3.84 units was significant at *P* < 0.05), and evaluation of goodness of fit (Fig. S2a through c) and individual plots (63, 64). Final model performance was evaluated using visual predictive checks (63–65), and parameter precision was determined using sampling importance resampling (66). Whenever no effect of clofazimine co-administration was found, *a posteriori* stochastic simulation and estimation (67) was performed to estimate the power of our sample size. Briefly, each final model was modified by adding a 30% effect of clofazimine on clearance and used to simulate 1,000 replicates of the study data set. The input model (with effect) and an alternative (without effect) were fitted to the simulated data sets, and the ΔOFV between the two models was evaluated for significance at *α* = 0.05. The power of the sample size was then derived from the percentage of simulations in which the clofazimine effect was statistically significant

## SUPPLEMENTAL MATERIAL

Supplemental material aac.01583-23-s0001.pdf Figures S1 to S3; Table S1.

ASM does not own the copyrights to Supplemental Material that may be linked to, or accessed through, an article. The authors have granted ASM a non-exclusive, world-wide license to publish the Supplemental Material files. Please contact the corresponding author directly for reuse.
