# Supplementary material for: Assessing potential drug-drug interactions between clofazimine and other frequently used agents to treat drug-resistant tuberculosis
Source: Antimicrob Agents Chemother. 2024 Apr 10;68(5):e01583-23. doi: 10.1128/aac.01583-23 (PMC11064479; doi:10.1128/aac.01583-23)
Supplement: Supplemental material — Figures S1 to S3; Table S1. [file aac.01583-23-s0001.pdf]

Assessing potential drug-drug interactions between clofazimine and other frequently used agents to treat drug-resistant tuberculosis.

Supplementary materials

Methods

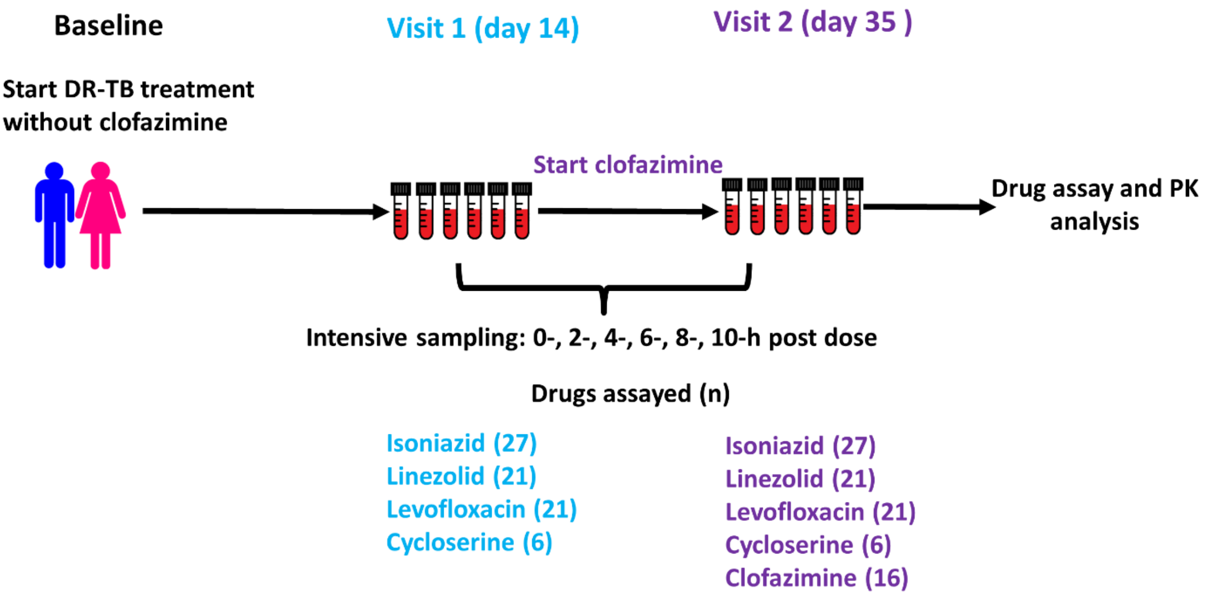

Figure S1: A schematic representation of study protocol showing the different study visits and what drugs were assayed from samples collected during the visits.

<u>Drug assays.</u>

Table S1: A table showing the inter-day accuracy, precision ranges and lower limits of quantification of the pharmacokinetic analysis methods for the assayed drugs. LLOQ; lower limit of quantification.

Drug analyzed	Range of inter-day accuracy (%)	Range of precision (%)	LLOQ (mg/L)
Clofazimine	102 – 103	2.50 – 5.00	0.00781
Isoniazid	95.3 – 100	5.70 – 6.50	0.105
Linezolid	90.1 – 98.1	3.50 – 3.60	0.100
Levofloxacin	94.5 – 98.6	2.40 – 3.00	0.0781
Cycloserine	89.9 – 97.0	3.30 – 15.3	0.313

Results

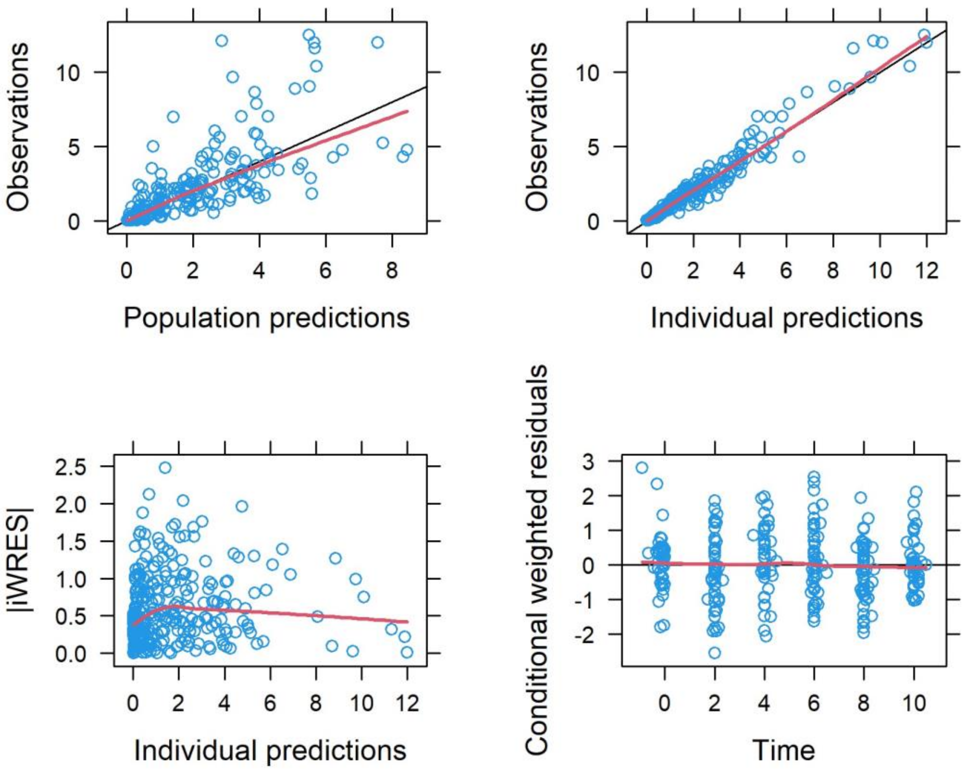

Figure S2a: Basic goodness-of-fit plots for the **Isoniazid** model. Scatter plot of (**top left**) observed concentration vs. population predicted values, (**top right**) observed concentration vs. individual predicted values, (**bottom left**) Individual weighted residuals vs. individual predictions and (**bottom right**) Conditional weighted residual vs. time. Each circle represents an individual data point from the respective analysis dataset.

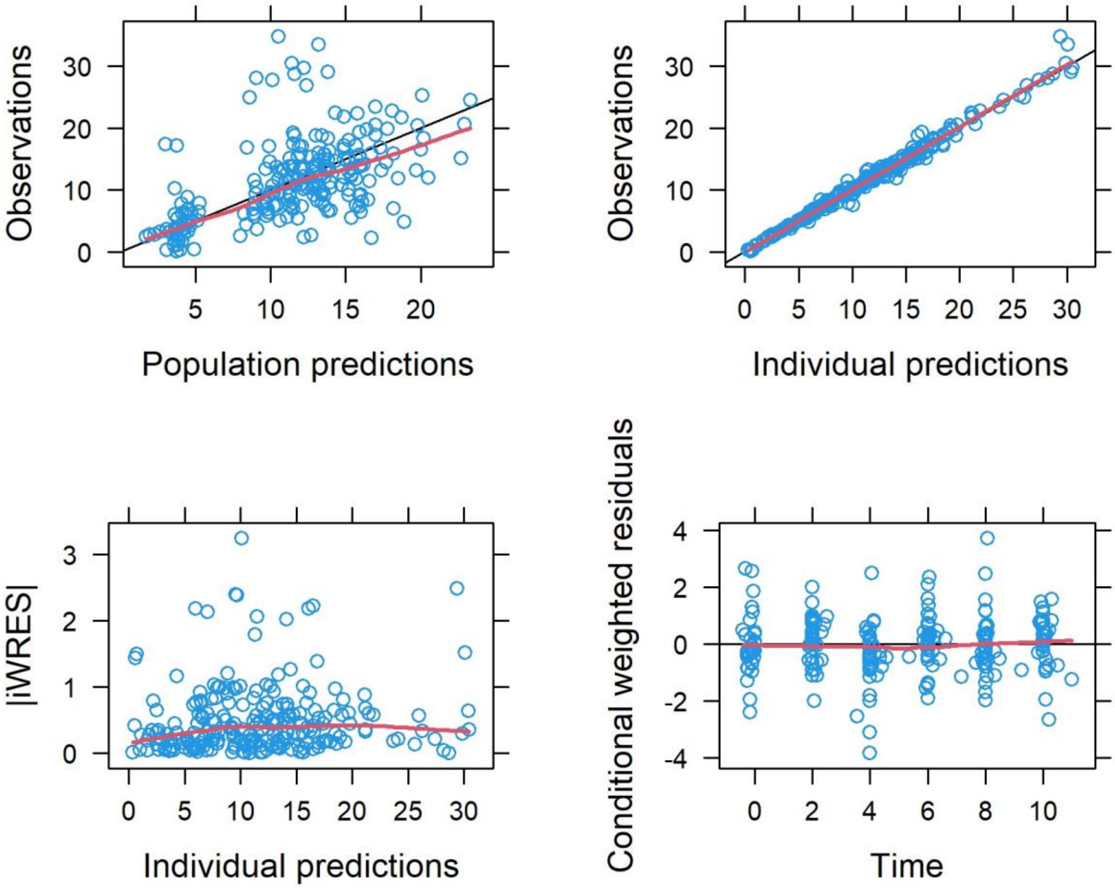

Figure S2b: Basic goodness-of-fit plots for the **linezolid** model. Scatter plot of (**top left**) observed concentration vs. population predicted values, (**top right**) observed concentration vs. individual predicted values, (**bottom left**) Individual weighted residuals vs. individual predictions and (**bottom right**) Conditional weighted residual vs. time. Each circle represents an individual data point from the respective analysis dataset.

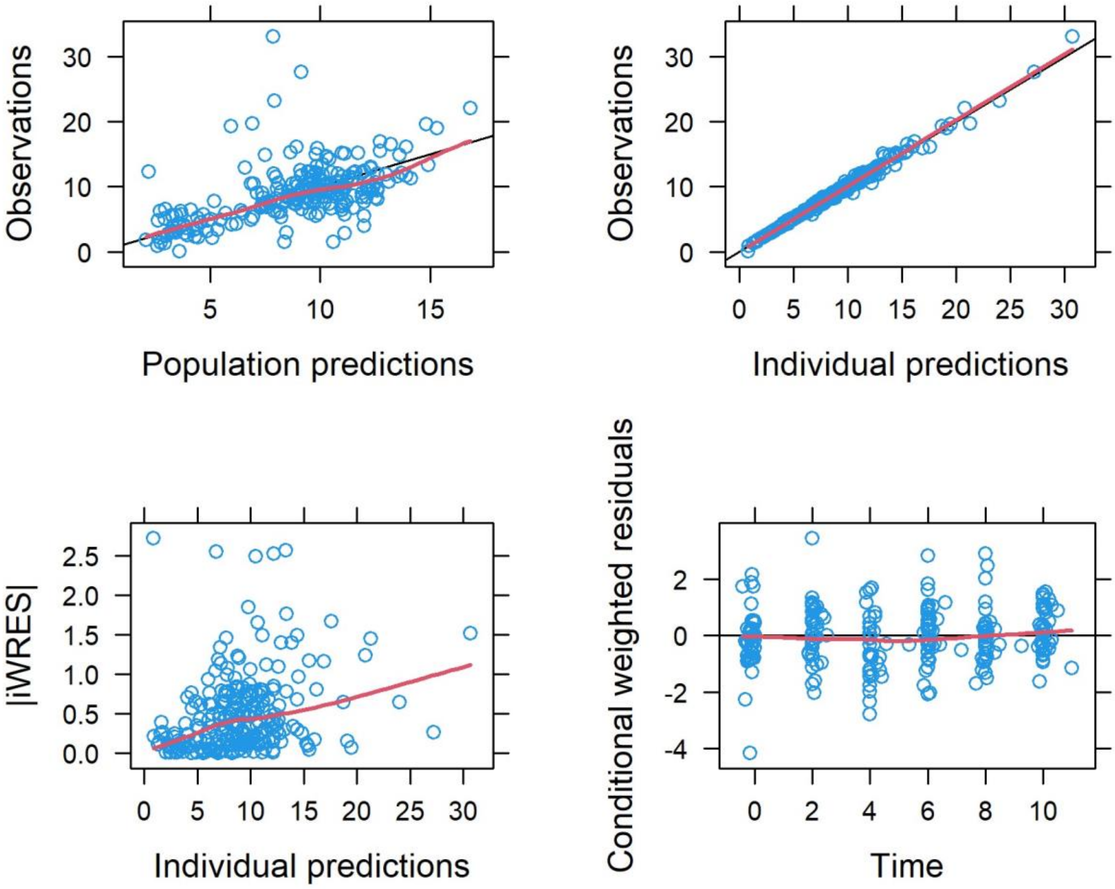

Figure S2c: Basic goodness-of-fit plots for the **levofloxacin** model. Scatter plot of (**top left**) observed concentration vs. population predicted values, (**top right**) observed concentration vs. individual predicted values, (**bottom left**) Individual weighted residuals vs. individual predictions and (**bottom right**) Conditional weighted residual vs. time. Each circle represents an individual data point from the respective analysis dataset.

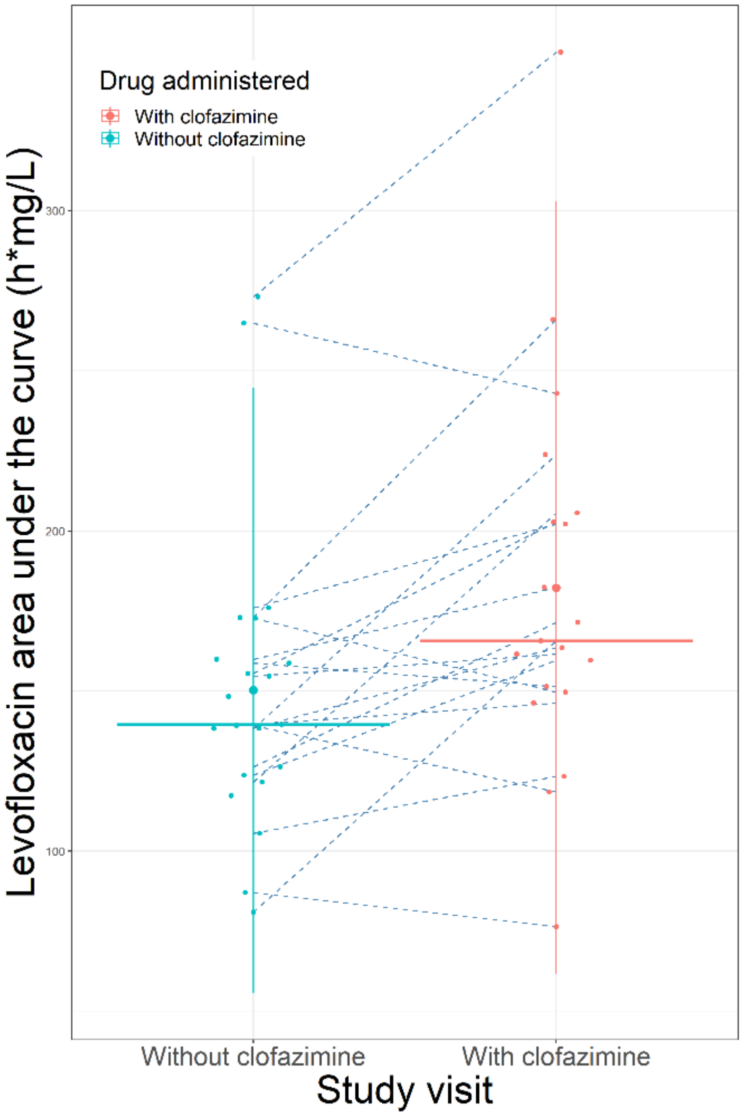

Figure S3: A bee swarm plot showing the change in levofloxacin area under the curve of participants before and after clofazimine was added to their drug-resistant TB regimen. Shrinkage in apparent between occasion bioavailability and between subject variability in clearance were 43% and 12% respectively.

References

1. Abdelwahab MT, Wasserman S, Brust JCM, Gandhi NR, Meintjes G, Everitt D, et al. Clofazimine pharmacokinetics in patients with TB: Dosing implications. Journal of Antimicrobial Chemotherapy. 2020 Nov 1;75(11):3269–77.

2. Abdelwahab MT, Leisegang R, Dooley KE, Mathad JS, Wiesner L, Mcilleron H, et al. Population Pharmacokinetics of Isoniazid, Pyrazinamide, and Ethambutol in Pregnant South African Women with Tuberculosis and HIV [Internet]. 2020. Available from: https://journals.asm.org/journal/aac

3. Abdelwahab MT, Wasserman S, Brust JCM, Dheda K, Wiesner L, Gandhi NR, et al. Linezolid Population Pharmacokinetics in South African Adults with Drug-Resistant Tuberculosis. 2021; Available from: https://doi.org/10.1128/AAC

4. Denti P, Garcia-Prats AJ, Draper HR, Wiesner L, Winckler J, Thee S, et al. Levofloxacin population pharmacokinetics in south african children treated for multidrug-resistant tuberculosis. Antimicrob Agents Chemother. 2018 Feb 1;62(2).

5. Court R, Wiesner L, Stewart A, De Vries N, Harding J, Maartens G, et al. Steady state pharmacokinetics of cycloserine in patients on terizidone for multidrug-resistant tuberculosis. International Journal of Tuberculosis and Lung Disease. 2018 Jan 1;22(1):30–3.
